# Transferrin combined with alanine aminotransferase and body mass index improves non-invasive diagnosis of metabolic dysfunction-associated steatohepatitis

**DOI:** 10.1530/EC-25-0591

**Published:** 2025-11-25

**Authors:** Xinyi Wang, He He, Libo Liang

**Affiliations:** ^1^ Department of Laboratory Medicine, West China Hospital, Sichuan University, Chengdu, PR China; ^2^ Sichuan Clinical Research Center for Laboratory Medicine, Chengdu, Sichuan, PR China; ^3^ Clinical Laboratory Medicine Research Center of West China Hospital, Chengdu, Sichuan, PR China; ^4^ Department of Laboratory Medicine, The Second People’s Hospital of Yibin, Yibin, Sichuan, PR China; ^5^ General Practice Medical Center, West China Hospital, Sichuan University, Chengdu, PR China

**Keywords:** transferrin, MASLD, MASH, BMI, non-invasive, diagnosis

## Abstract

**Background:**

Metabolic dysfunction-associated steatotic liver disease (MASLD) is a leading cause of chronic liver disease globally, with over 20% of patients progressing to metabolic dysfunction-associated steatohepatitis (MASH), which carries a high risk of cirrhosis and hepatocellular carcinoma. While liver biopsy remains the gold standard for MASH diagnosis, its invasiveness and limitations necessitate reliable noninvasive alternatives. This study aimed to develop a cost-effective biomarker panel using routine laboratory tests to distinguish MASLD severity stages.

**Methods:**

We conducted a single-center, retrospective study of 209 biopsy-proven MASLD patients, stratified by NAS: simple steatosis (NAS <3, *n* = 40), borderline (NAS 3–4, *n* = 120), and definitive MASH (NAS ≥5, *n* = 49). Clinical, biochemical, hematologic parameters and metabolic markers were analyzed. Logistic regression and ROC curves assessed diagnostic performance.

**Results:**

Key findings revealed progressive increases in BMI, ALT, AST, and transferrin (Tf) levels with disease severity. Multivariate logistic regression identified ALT and Tf as independent predictors for borderline and MASH. Notably, ALT showed superior diagnostic performance for distinguishing simple MASLD with borderline (AUC 0.763), while Tf was most effective for MASH detection (AUC 0.723). A combined model integrating BMI, ALT, and Tf demonstrated excellent diagnostic accuracy for borderline (AUC 0.840) and MASH (AUC 0.805).

**Conclusion:**

Our study highlights that a simple, cost-effective panel of routinely available biomarkers (BMI, ALT, and Tf) can effectively stratify MASLD progression, offering a practical alternative to invasive diagnostics. This approach enhances early MASH detection, facilitating timely clinical intervention.

## Introduction

The global prevalence of metabolic dysfunction-associated steatotic liver disease (MASLD) is rising, establishing it as a leading cause of chronic liver disease worldwide (1, 2). A significant proportion of individuals progress from simple hepatic steatosis to metabolic dysfunction-associated steatohepatitis (MASH) (3). Over time, the patients develop into cirrhosis, hepatocellular carcinoma (4), and even extrahepatic malignancies (5), imposing a substantial clinical and economic burden (6). Therefore, we should pay more attention to monitor the MASLD progression, particularly the transition to MASH.

Currently, liver biopsy remains the gold standard for MASH diagnosis, and relying on histopathological assessment (7). However, its invasive nature, sampling variability (8), procedural risks, and high costs limit its utility in routine clinical practice (9). These constraints underscore the need for reliable, noninvasive alternatives. Several noninvasive approaches have emerged for MASLD monitoring and MASH detection, such as magnetic resonance imaging–based techniques (10, 11), serum metabolomic panels (12, 13, 14), and composite scores integrating clinical, biochemical, and imaging parameters (15, 16). Besides, biomarkers such as cytokeratin-18 (CK-18) have also been proposed as surrogates for biopsy (17, 18). While some show promise, they can be expensive, not readily available, or require further validation. Furthermore, emerging evidence suggests a role for iron metabolism dysregulation in the pathogenesis and progression of MASLD (19), highlighting its potential as a source of biomarkers.

Given the need for cost-effective and accessible tools, we aimed to identify high-performance biomarkers derived from routine laboratory tests by analyzing standard biochemical and hematological parameters across histologically confirmed MASLD and MASH cohorts to evaluate their diagnostic utility.

## Materials and methods

### Study population

**Figure 1:**
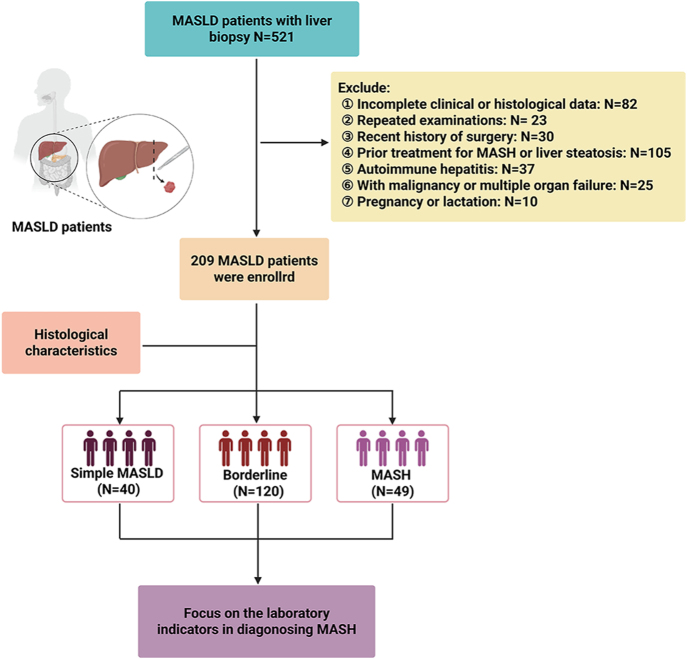
The study flowchart.

This retrospective analysis utilized a convenience sample of 521 MASLD patients with available liver biopsy data at the hospital from May 2023 to June 2024. The diagnosis of MASLD required imaging or histologic evidence of ≥5% hepatic steatosis, concomitant with at least one of the following metabolic criteria: overweight/obesity, type 2 diabetes, or metabolic dysregulation (20). The exclusion criteria were as follows: i) patients lacking basic information or histological data (*n* = 82); ii) patients with repeated examinations (only the first results were retained) (*n* = 23); iii) patients who had received surgery recently (*n* = 30); iv) patients receiving pharmacotherapies for MASH or medications for improving liver steatosis (for example, vitamin E, amiodarone, or pioglitazone) were excluded (*n* = 105); v) patients who have diagnosed with autoimmune hepatitis (*n* = 37); vi) patients with malignant tumors or multiple organ failure (*n* = 25); and vii) women during pregnancy and lactation (*n* = 10). In total, 209 patients (40.11%) were eventually included in our research (Fig. 1). Meanwhile, all data were obtained before puncture. This study was performed in line with the principles of the Declaration of Helsinki. Approval was granted by the Ethics Committee (NO: 20131232). Informed consent was obtained from all individual participants included in the study.

### Data collection

Comprehensive clinical data were collected, including demographic characteristics (age, sex), anthropometric measurements (height, weight, body mass index (BMI; calculated as weight in kilograms divided by height in meters squared)), blood pressure parameters (systolic (SBP) and diastolic (DBP)), alcohol consumption history, and current/past medical history. Histological characteristics included steatosis grades, lobular inflammation grades, ballooning grades, and the levels of liver fibrosis. Laboratory analyses included 35 biochemical, immunological, and hematological parameters: alanine aminotransferase (ALT), aspartate aminotransferase (AST), total bilirubin (TBIL), direct bilirubin (DBIL), total protein, albumin, globulin (GLB), uric acid (UA), UREA, CREA, estimated glomerular filtration rate (eGFR), fasting plasma glucose (FPG), total cholesterol (TC), glycated hemoglobin (HbA1c), apolipoprotein A (apo A), apolipoprotein B (apo B), triglycerides (TG), high-density lipoprotein cholesterol (HDL-C), low-density lipoprotein cholesterol (LDL-C), red blood cell (RBC), hemoglobin (HGB), hematocrit (HCT), mean corpuscular volume (MCV), mean corpuscular hemoglobin (MCH), mean corpuscular hemoglobin concentration (MCHC), platelet (PLT), white blood cell count (WBC), neutrophil (NEUT), lymphocyte (LYMPH), monocyte (MONO), basophil (BASO) and eosinophil (EO), and transferrin (Tf).

### Definition

NAS was calculated as the sum of the three histological components, including steatosis (grade 0–3), ballooning (grade 0–2), and lobular inflammation (grade 0–3). Fibrosis was graded from 0 to 4. We defined MASH with NAS ≥5, including NAS scoring at least 1 point in each category of steatosis, lobular inflammation, and ballooning. Borderline MASH was defined as NAS of 3–4, while NAS <3 and no fibrosis were diagnosed with MASLD (simple steatosis).

### Statistical analysis

Statistical analysis was performed with SPSS version 26.0 and GraphPad Prism software (version 8.0). Continuous variables were represented by mean ± standard deviation (normal distribution) or median and interquartile range (non-normal distribution), while categorical variables were expressed as percentages. Mann–Whitney U test or *t*-test were used for analyzing continuous variables, and the chi-squared test was used for categorical variables. The relationships between Tf and other parameters were analyzed by R software (version 3.6.0), and receiver operating characteristic (ROC) curves were utilized to evaluate the diagnostic efficacy of the parameters. All statistical tests are bilateral probability tests, with a *P*-value of <0.05 indicating statistical significance.

## Results

### Characteristics of the patients

This cross-sectional study included a total of 209 patients who were diagnosed with MASLD. The median age was 33 years (range: 18–61 years). Most of the patients were under the age of 50 years (93%) and had no drinking (88%) and DM history (82.3%). The histological characteristics of participants are indicated in the Table 1. A total of 159 (76.1%) patients had varying levels of liver fibrosis. The NAS grades were classified as simple MASLD in 40 patients (19.2%), borderline in 120 patients (57.4%), and MASH in 49 patients (23.4%).

**Table 1:** Characteristics of the study patients.

Characteristics	Value
Number of patients	209
Age, median	33 (18, 61)
<50	195 (93%)
≥50	14 (7%)
Sex	
Male	84 (40%)
Female	125 (60%)
Drinking	
No	184 (88%)
Yes	25 (12%)
DM history	
No	37 (17.7%)
Yes	172 (82.3%)
Steatosis grades	
1	147 (70.33%)
2	41 (19.62%)
3	21 (10.05%)
Lobular inflammation grades	
0	1 (0.47%)
1	164 (78.47%)
2	42 (20.10%)
3	2 (0.96%)
Ballooning grades	
0	9 (4.31%)
1	175 (83.73%)
2	25 (11.96%)
Fibrosis stages	
0	50 (23.9%)
1	102 (48.8%)
2	31 (14.8%)
3	16 (7.7%)
4	10 (4.8%)
NAS grades	
<3	40 (19.2%)
≥3, <5	120 (57.4%)
≥5	49 (23.4%)

### Clinical and laboratory characteristics of the patients stratified by NAS grades

Compared to simple MASLD, both borderline and MASH groups demonstrated significantly higher BMI values (*P* < 0.001) and more pronounced hepatic and metabolic dysfunction (Table 2). Borderline cases showed significantly elevated serum levels of TBA, ALT, AST, and Tf than simple MASLD (all *P* < 0.05). Furthermore, MASH patients exhibited significantly higher BMI, ALT, AST, UA, TG, FPG, HbA1c, EO%, and Tf levels compared to borderline cases (all *P* < 0.05).

**Table 2:** Comparison of clinical and laboratory characteristics among the three groups.

	Simple MASLD (40)	Borderline (120)	MASH (49)	*P*
Male (*n*, %)	15 (37.5%)	51 (42.5%)	18 (39.1%)	0.383
DM2 (*n*, %)	18 (45%)	60 (50%)	22 (44.9%)	0.169
Drinking (*n*, %)	2 (5.0%)	14 (11.7%)	9 (18.4%)	0.02
Age (years)	34 (29, 38)	33 (28, 39)	32 (29, 37.5)	0.477
BMI (kg/m^2^)	33.30 (32.09, 37.9)*	**36.33 (33.23, 39.4)**	39.2 (36.25, 43.44)*	**<0.001**
SBP (mmHg)	128.5 (120.25, 135.0)	132.5 (120.75, 144)	130 (123.25, 140)	0.208
DBP (mmHg)	84 (77, 89)	87 (80, 93)	86 (79, 93)	0.34
TBIL (μmol/L)	9.8 (7.2, 11.9)	10.4 (7.95, 13.7)	11.5 (8.7, 16.7)	0.554
DBIL (μmol/L)	2.4 (1.7, 3.1)	2.7 (2.0, 3.5)	3.1 (2.25, 4.0)	0.5
TBA (μmol/L)	2.3 (1.67, 3.6)*	3.3 (1.85, 5.0)	2.6 (1.55, 4.50)	0.027
ALT (IU/L)	20 (15, 26)*	**39 (25.5, 54.5)**	60 (39.5, 93)*	**<0.001**
AST (IU/L)	18 (15, 24)*	**25 (19.5, 25)**	36 (27, 57)*	**<0.001**
TP (g/L)	74.2 (71.1, 76.2)	73.2 (70.15, 76.4)	76 (72.7, 79.05)	0.001
ALB (g/L)	46.3 (44.2, 47.7)	45.9 (43.5, 48.2)	47 (44.9, 48.65)	0.071
GLB (g/L)	28.5 (25.3, 30.3)	26.8 (24.4, 29.8)	28.4 (26.3, 30.8)	0.049
UREA (mmol/L)	4.5 (3.5, 5.7)	4.2 (3.55, 5.10)	4.1 (3.6, 5.05)	0.762
CREA (μmol/L)	61 (54, 72)	62 (54, 76)	63.5 (57, 75)	0.734
eGFR (mL/min/1.73 m^3^)	113.53 (94.13, 122.1)	115.37 (107.56, 123.11)	116.50 (106.59, 124.07)	0.751
UA (μmol/L)	357 (309, 415)*	406 (321, 491.5)	440.5 (365.25, 521)*	0.012
TG (mmol/L)	1.32 (1.13, 1.73)	1.58 (1.14, 2.31)	2.05 (1.39, 2.89)*	0.036
TC (mmol/L)	4.64 (4.19, 5.42)	4.66 (4.05, 5.20)	4.85 (4.21, 5.56)	0.87
HDL-c (mmol/L)	1.14 (1.02, 1.38)	1.04 (0.95, 1.24)	1.06 (0.92, 1.22)	0.067
LDL-c (mmol/L)	3.07 (2.51, 3.44)	2.96 (2.49, 3.45)	3.03 (2.43, 3.63)	0.736
APOA (g/L)	1.25 (1.15, 1.43)	1.24 (1.13, 1.38)	1.39 (1.17, 1.42)	0.585
APOB (g/L)	1.02 (0.92, 1.21)	1.07 (0.93, 1.26)	1.13 (0.90, 1.33)	0.395
A/B	1.21 (1.01, 1.57)	1.22 (0.97, 1.49)	1.15 (0.97, 1.38)	0.687
FPG (mmol/L)	5.46 (5.21, 6.01)	5.69 (5.29, 6.57)	6.81 (5.85, 8.08)*	<0.001
HbA1c (%)	5.7 (5.5, 6.08)	5.8 (5.5, 6.35)	6.5 (6.0, 7.35)*	<0.001
RBC (10^12^/L)	4.84 (4.56, 5.15)	5.02 (4.57, 5.35)	5.19 (4.95, 5.61)	0.005
HGB (g/L)	137 (134, 150)	144 (134, 155.5)	150 (138.5, 164)	0.005
HCT	0.43 (0.41, 0.46)	0.45 (0.41, 0.48)	0.46 (0.43, 0.50)	0.034
MCV (fL)	89.8 (86.3, 91.9)	89.6 (87.4, 92.25)	89.9 (86.6, 93.6)	0.955
MCH (PG)	29 (27.9, 30.1)	29.3 (28.15, 30.3)	29.2 (28.5, 30.5)	0.379
MCHC (g/L)	323 (319, 329)	324 (317, 332)	327 (320, 335.5)	0.204
PLT (10^9^/L)	269 (225, 330)	271 (211.5, 318.5)	273 (221, 320.5)	0.897
WBC (10^9^/L)	8.62 (6.74, 9.93)	8.26 (6.48, 9.89)	7.93 (7.37, 9.6)	0.323
NEUT (%)	64 (57.7, 70.9)	63.6 (58.1, 72.6)	61.7 (54.6, 68.6)	0.585
LYMPH (%)	25.7 (21.9, 33.0)	27.6 (20.6, 32.15)	29.5 (24.6, 36.1)	0.661
MONO (%)	5.6 (4.5, 7.0)	5.4 (4.25, 6.65)	6.0 (5.1, 7.2)	0.218
EO (%)	1.1 (0.6, 2.0)	1.4 (0.75, 2.20)	2.1 (1.15, 2.6)*	0.032
BASO (%)	0.4 (0.2, 0.6)	0.40 (0.30, 0.60)	0.6 (0.4, 0.7)	0.037
Tf (mg/dL)	104 (50.8, 161)*	**177 (91.25, 287)**	345 (189.9, 624)*	**<0.001**

* Bold indicates statistical significance; *P* < 0.05, compared to borderline group.

BMI, body mass index; SBP, systolic blood pressure; DPB, diastolic blood pressure; ALT, alanine aminotransferase; AST, aspartate aminotransferase; TBIL, total bilirubin; DBIL, direct bilirubin; TP, total protein; ALB, albumin; GLB, globulin; UA, uric acid; CREA, creatinine; eGFR, estimated glomerular filtration rate; FPG, fasting plasma glucose; TC, total cholesterol; HbA1c, glycated hemoglobin; APOA, apolipoprotein A; APOB, apolipoprotein B; TG, triglycerides; HDL-C, high-density lipoprotein cholesterol; LDL-C, low-density lipoprotein cholesterol; RBC, red blood cell; HGB, hemoglobin; HCT, hematocrit; MCV, mean corpuscular volume; MCH, mean corpuscular hemoglobin; MCHC, mean corpuscular hemoglobin concentration; PLT, platelet; WBC, white blood cell count; NEUT, neutrophil; LYMPH, lymphocyte; MONO, monocyte; BASO, basophil; EO, eosinophil; Tf, transferrin.

### Association between markers, and risk of borderline and MASH

**Figure 2:**
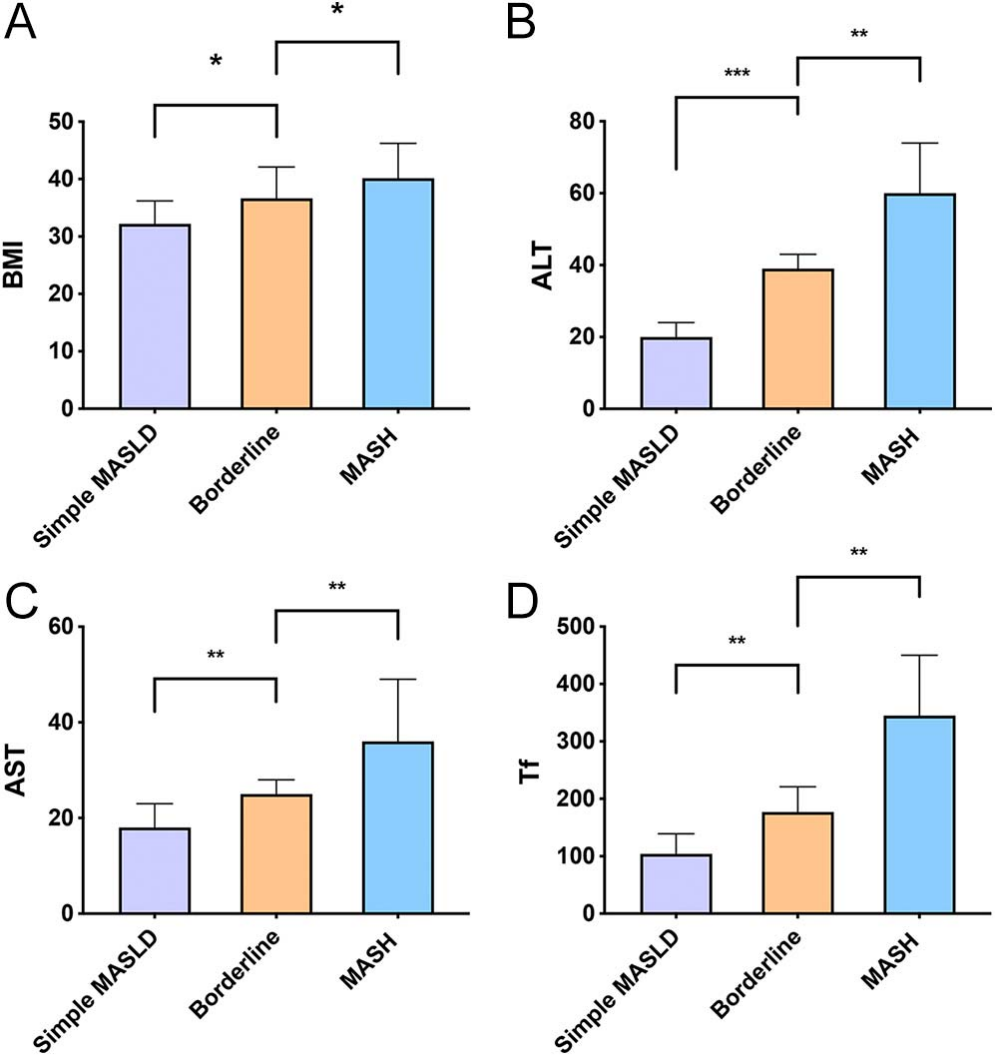
The distribution of four markers in the simple MASLD group, borderline, and MASH groups. (A) BMI distribution in three groups; (B) ALT distribution in three groups; (C) AST distribution in three groups; (D) Tf distribution in three groups. *means *P* < 0.05, **means *P* < 0.01, ***means *P* < 0.001.

Figure 2 illustrates the progressive increase in BMI, ALT, AST, and Tf levels across the simple MASLD, borderline, and MASH groups (all *P* < 0.05). Multivariate logistic regression analyses, adjusted for age and sex, consistently identified both ALT and Tf as independent predictors for MASLD progression. Elevated levels of these markers were significantly associated with an increased risk of both borderline and MASH. The full details of these analyses, including odds ratios and confidence intervals, are presented in Supplementary Tables S1 and S2 (see section on Supplementary materials given at the end of the article).

### Correlations between Tf and metabolic parameters

**Figure 3:**
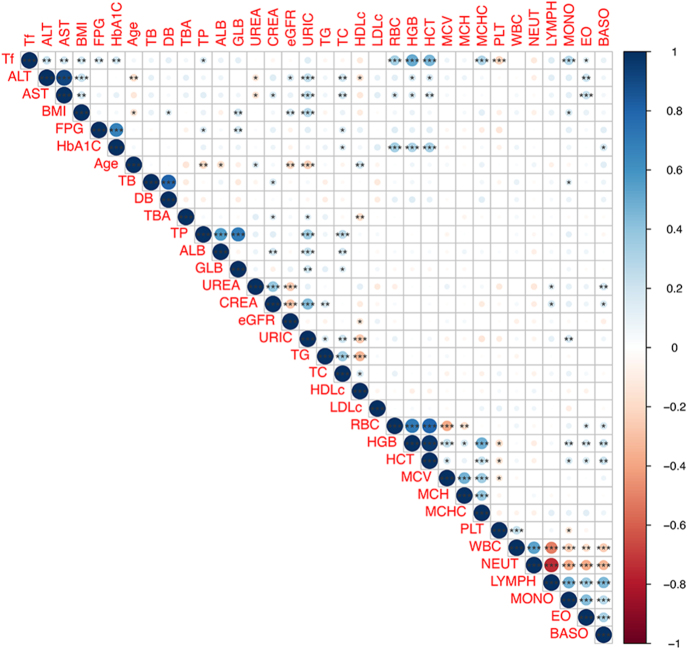
Visualization of the correlation of enrolled laboratory indicators. *means *P* < 0.05, **means *P* < 0.01, ***means *P* < 0.001.

Rank correlation analysis demonstrated significant positive associations between serum Tf levels and BMI, ALT, AST, FPG, and HbA1c (all *P* < 0.01), while showing an inverse correlation with PLT (*P* < 0.001) (Fig. 3). These findings indicate moderate but significant relationships between transferrin and both metabolic derangements and hepatic injury markers.

### Diagnostic performance of biomarkers for MASLD severity

**Figure 4:**
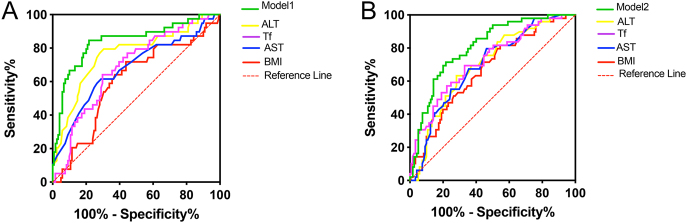
ROC curves for diagnosis in different groups: (A) the simple MASLD group and the borderline group; (B) the borderline group and the MASH group.

As shown in Fig. 4, the performance of individual biomarkers varied across disease stages. ALT proved superior for identifying borderline (AUC 0.763; 95% CI: 0.671–0.855) compared to Tf 0.686 (95% CI: 0.594–0.778), AST 0.679 (95% CI: 0.576–0.782), and BMI 0.603 (95% CI: 0.499–0.706) (all *P* < 0.001). While Tf was the strongest single predictor for MASH (AUC 0.723; 95% CI: 0.639–0.807), the AUCs were followed by ALT 0.698 (95% CI: 0.615–0.781), AST 0.695 (95% CI: 0.611–0.778), and BMI 0.668 (95% CI: 0.581–0.756). However, the combined diagnostic models substantially outperformed any single marker. The model for borderline (Tf, ALT, BMI) achieved an AUC of 0.840 (95% CI: 0.761–0.920), with 84.6% sensitivity and 78.3% specificity, and the model for MASH reached an AUC of 0.805 (95% CI: 0.736–0.874), with 73.5% sensitivity and 73.3% specificity, underscoring the synergistic value of integrating these parameters.

## Discussion

Once MASLD is diagnosed, clinicians need to assess its severity and determine whether the patient is at risk for MASH (21). Our aim was to find noninvasive biomarkers that can be used as an alternative to puncture by classifying pathological results and then comparing the differences in routine clinical indicators. In the current study, the levels of BMI, ALT, AST, and Tf positively correlated with the progression from MASLD to MASH. When distinguishing between simple MASLD and borderline, ALT performed better. Notably, Tf performed better when distinguishing between borderline and MASH. Our study demonstrates that a combination of simple, clinically accessible biomarkers – BMI, ALT, AST, and Tf – can effectively distinguish between MASLD disease stages.

The main feature of MASLD is the accumulation of fat (1), and the development is closely related to the inflammatory response (22). Obesity, a key driver of metabolic dysfunction, exacerbates hepatic lipid accumulation and insulin resistance, promoting inflammation and fibrosis (23). Notably, adipose tissue dysfunction in obesity leads to increased release of free fatty acids and proinflammatory cytokines, further fueling hepatic inflammation and oxidative stress. Emerging evidence suggests the progressive form of MASLD is MASH (3). MASH is frequently linked to aberrant iron metabolism, and ferroptosis has been identified as a key mechanism driving hepatocyte injury, inflammation, and fibrosis in MASH (24, 25). Obesity can exacerbate oxidative stress and lipid peroxidation (26), thereby intensifying cell damage and inflammation (27). Clinical studies have consistently demonstrated a positive correlation between BMI and systemic inflammatory markers, suggesting that adiposity-driven inflammation worsens with increasing obesity severity (28). Interestingly, some inflammatory markers were reduced in people who reduced BMI by not undergoing laparoscopic adjustable gastric banding (29). Our data align with these findings, showing that elevated BMI is associated with higher serum levels of AST and ALT, reinforcing the role of excessive adiposity in hepatocellular injury. However, the relationship between BMI and MASH is not strictly linear; some lean individuals also develop significant liver disease (30). Thus, while obesity remains a major risk factor for MASH, genetic and metabolic factors may also contribute beyond adiposity.

ALT and AST are well-established indicators of hepatocellular damage (31, 32). In this study, it was demonstrated that the concentrations of both ALT and AST were elevated to a greater extent in the MASH group when compared with patients in the simple MASLD and borderline groups. For identifying simple MASLD, ALT exhibited the highest diagnostic accuracy (AUC 0.763). This aligns with ALT’s established role as a sensitive marker of hepatocellular injury in early fatty liver disease. Furthermore, a gradual increase from MASLD to MASH was observed, thus indicating that ALT and AST play a pivotal role in the progression of MASLD. Elevated concentrations of AST and ALT in MASH patients are in alignment with the findings of other studies (33, 34). Nevertheless, only ALT is the independent risk factor in borderline and MASH. Besides, ALT showed a stronger association with steatosis progression than AST. This difference likely stems from the distinct tissue distributions of these enzymes. While ALT is predominantly localized in the liver, AST is more widely expressed in other tissues, including skeletal muscle, heart, kidney, and red blood cells. Several factors contribute to the preferential elevation of ALT in MASH. These include sustained hepatocyte injury, increased mitochondrial dysfunction, and enhanced oxidative stress, all of which are marks of progressive MASLD. The greater specificity of ALT for liver damage makes it a more reliable indicator of steatohepatitis compared to AST. These observations underscore the importance of distinguishing between ALT and AST when evaluating MASLD severity.

The diagnostic performance shifted when evaluating MASH, where Tf emerged as the superior single marker. This finding suggests Tf levels may become increasingly relevant as MASLD progresses to MASH. Tf is responsible for maintaining the balance between iron uptake, storage, and utilization (35). Iron is required for the growth and reproduction of most pathogenic bacteria, and Tf binds iron to inhibit the growth of pathogenic bacteria during early inflammation. One study showed bacterial molecules such as LPS can increase Tf levels and control the immune response (36). Zhao *et al.* showed that the levels of interleukin-6 and interferon-γ were positively associated with the Tf receptor (37). However, it is unregulated during the acute-phase response to infection (38). In severe inflammation, Tf releases iron, which leads to oxidative stress and exacerbates tissue damage and inflammation. Lymphocytes and macrophages need iron to support their proliferation and immune function (39). Tf provides iron efficiently, but an excessive immune response can lead to more severe inflammatory pathologies (40). This dysregulation of iron metabolism is common in patients with complex diseases involving chronic inflammation (41). A recent research study underscored the moonlighting function of Tf in lipid catabolism (42). We observed that higher Tf levels correlate with increased AST and ALT, reinforcing the link between iron metabolism dysregulation and hepatocellular injury. The liver is the primary organ that regulates iron homeostasis (43). When hepatocytes die, iron release increases, and more Tf is required, so it is accompanied by an increase in Tf. Since transferrin-bound iron can promote hepatic oxidative stress, its elevation may exacerbate liver damage, creating a vicious cycle of inflammation and fibrosis (44, 45). In addition, BMI modulated this relationship, with obese MASH patients showing the highest transferrin levels. Adipose tissue inflammation and hepatokine dysregulation may further disrupt iron metabolism, amplifying transferrin elevation in the context of metabolic syndrome.

Fibrosis-4 index (FIB-4) and fibrosis score (NFS) are the two most widely used noninvasive tools for screening advanced fibrosis in subjects with MASLD (46). They also apply to fibrosis screening for hepatic fibrosis in the general population (47). In our study, we paid more attention to inflammation levels during MASLD to MASH progression. We emphasize that while FIB-4 and NFS are excellent for fibrosis risk stratification, our panel offers a distinct advantage in MASH risk stratification. The true clinical value of our study lies in the combination models we developed: i) for borderline MAFLD, the optimal panel combining Tf, ALT, and BMI achieved excellent diagnostic accuracy (AUC 0.840), with balanced sensitivity (84.6%) and specificity (78.3%); ii) for MASH diagnosis, the combination model maintained strong performance (AUC 0.805) with consistent sensitivity (73.5%) and specificity (73.3%). While noninvasive biomarkers are increasingly used, combining these with metabolic parameters and iron-related markers could enhance diagnostic accuracy.

There are also some limitations in our study. First, it is a cross-sectional, retrospective, single-center study. Second, we lack an independent validation cohort. Consequently, future studies with larger sample sizes and a multicenter design are needed to validate these results.

We utilized only routine clinical tests, avoiding expensive or specialized biomarkers. This simple, effective diagnostic approach could facilitate earlier identification of patients progressing to MASH. Complex, expensive tests are always necessary for MASLD management. Our findings support the utility of optimized combinations of routine tests as practical diagnostic tools for fatty liver disease progression.

## Supplementary materials



## Declaration of interest

The authors declare that there is no conflict of interest that could be perceived as prejudicing the impartiality of the work reported.

## Funding

This work was supported by grants from the National Natural Science Foundation of China (grant No. 32271494 and 82302583), and the Sichuan Science and Technology Program (grant number 2025ZNSFSC1619).

## Author contribution statement

All authors contributed to the study conception and design. Material preparation, data collection, analysis, and manuscript writing were performed by Wang XY. He H Liang LB was responsible for the conceptualization and visualization of the study, and writing, reviewing, and editing. All authors read and approved the final manuscript.

## Ethics approval statement

This study was performed in line with the principles of the Declaration of Helsinki. Approval was granted by the Ethics Committee of West China Hospital, Sichuan University (NO.: 20131232). Informed consent was obtained from all individual participants included in the study.
