# Supplementary material for: Transferrin combined with alanine aminotransferase and body mass index improves non-invasive diagnosis of metabolic dysfunction-associated steatohepatitis
Source: Endocr Connect. 2025 Nov 25;14(11):e250591. doi: 10.1530/EC-25-0591 (PMC12665513; doi:10.1530/EC-25-0591)
Supplement: Supplementary file 1 [file supplementary_materials.pdf]

Supplementary Table 1 Univariate analyses of clinical parameters in borderline

Variables	β	S.E.	Wald	*P*	OR	95%CI	
						Lower	Upper
Age (≥50 vs <50)	1.366	1.132	1.456	0.228	0.255	0.028	2.346
Gender (male vs female)	0.941	0.576	2.673	0.102	2.563	0.829	7.922
Smoking (yes vs no)	-0.204	0.917	0.05	0.824	0.815	0.135	4.914
BMI	-0.005	0.043	0.013	0.908	0.995	0.915	1.082
ALT	0.057	0.017	11.916	0.001	1.059	1.025	1.094
Tf	0.005	0.002	6.884	0.009	1.005	1.001	1.009

BMI, body mass index; ALT, alanine aminotransferase; Tf, transferrin.

Supplementary Table 2 Univariate analyses of clinical parameters in MASH

Variables	β	S.E.	Wald	*P*	OR	95%CI	
						Lower	Upper
Age (≥50 vs <50)	-0.576	0.72	0.64	0.424	0.562	0.137	2.305
Gender (male vs female)	0.156	0.427	0.134	0.714	1.169	0.507	2.698
Smoking (yes vs no)	-0.198	0.545	0.132	0.716	0.820	0.282	2.389
BMI	0.101	0.036	7.628	0.060	1.106	1.03	1.188
ALT	0.012	0.005	4.942	0.026	1.012	1.001	1.023
Tf	0.004	0.001	17.422	0. 000	1.004	1.002	1.005
